# Special Issue “Emerging Virus Infections in Adverse Pregnancy Outcomes”

**DOI:** 10.3390/v14020285

**Published:** 2022-01-29

**Authors:** Léo Pomar, David Baud

**Affiliations:** 1 Materno-Fetal and Obstetrics Research Unit, Department Woman-Mother-Child, Lausanne University Hospital, University of Lausanne, 1011 Lausanne, Switzerland; 2 School of Health Sciences (HESAV), University of Applied Sciences and Arts Western Switzerland, 1011 Lausanne, Switzerland

Dear contributors and readers,

In this 2021 edition of the Special Issue “Emerging Virus Infections in Adverse Pregnancy Outcomes”, we have received and published some very relevant studies on these topics. Regarding congenital Zika infections, a mouse model confirmed that the embryological stage at the time of congenital infection was a determining factor for adverse outcomes in infected fetuses and that maternal neutralizing antibodies could protect the offspring from neonatal death after congenital infection. The results of the International Zika Virus in Pregnancy Registry showed that the risk of infection was lower among pregnant women who travelled in endemic areas compared to residents and was related to the presence of ongoing outbreaks and stay duration. In this registry, adverse perinatal outcomes were observed in 8.3% of infected travelers and 12.7% of infected residents. 

We also received several high-quality papers on SARS-CoV-2 infections during pregnancy. Rare but dramatic cases of placental and fetal infection, resulting in fetal or neonatal death or severe neonatal morbidity, were reported. The analysis of infected placentas revealed extensive and multifocal chronic intervillositis, as well as malperfusion, potentially causing leucomalacia in neonates. Fetal and early neonatal infections were also confirmed in several cases published in this issue, and a decrease in fetal movements was described as a warning sign for adverse perinatal outcomes following maternal SARS-CoV-2 infection. Concerning maternal outcomes, a cohort study from the Spanish Obstetric Emergency Group and a Brazilian cohort of pregnant individuals with high rates of comorbidities presented the risks of maternal and obstetrical adverse outcomes related to COVID-19 during pregnancy: death, pneumonia, ICU admission, iatrogenic prematurity, venous thrombotic events, severe pre-eclampsia, fetal, and neonatal death. We are also very grateful for a study based on a cohort in Mexico that analyzed the endothelial response to COVID-19 during pregnancy through the modification of the Soluble Fms-like Tyrosine Kinase-1/Angiotensin-II ratio. Finally, a Swiss study investigated the willingness of pregnant individuals to receive the SARS-CoV-2 vaccine and potential barriers to their immunization. 

Overall, we would like to thank all the researchers and clinicians who contributed to this Special Issue, and we hope to work with them again for future editions. We will continue this Special Issue with a 2022 edition on the same topics, in which we would also like to give more space to other emerging and endemic viruses that can complicate pregnancies.

